# Experimental acute lung injury induces multi-organ epigenetic modifications in key angiogenic genes implicated in sepsis-associated endothelial dysfunction

**DOI:** 10.1186/s13054-015-0943-4

**Published:** 2015-05-11

**Authors:** Karol Bomsztyk, Daniel Mar, Dowon An, Roya Sharifian, Michal Mikula, Sina A Gharib, William A Altemeier, W Conrad Liles, Oleg Denisenko

**Affiliations:** UW Medicine South Lake Union, University of Washington, 850 Republican Street, 98109 Seattle, WA USA; Department of Medicine, University of Washington, 850 Republican Street, 98195 Seattle, WA USA; Center for Lung Biology, University of Washington, 850 Republican Street, 98109 Seattle, WA USA

## Abstract

**Introduction:**

The Tie2/angiopoietin (Tie2/Ang) and vascular endothelial growth factor receptor-ligand systems (VEGFR/VEGF) are recognized to play important roles in the regulation of microvascular endothelial function. Downregulation of these genes during sepsis has been implicated in the pathogenesis of sepsis-related microvascular leak and multiple organ dysfunction syndrome. Mechanisms responsible for dysregulation of angiogenic genes in sepsis are poorly defined.

**Methods:**

Western blot, reverse transcription-polymerase chain reaction, and multiplex chromatin immunoprecipitation platform (Matrix ChIP) were used to investigate serum albumin leak, changes in gene expression, and associated epigenetic alterations in a murine model of acute lung injury-induced sepsis (ALI-sepsis).

**Results:**

Experimental ALI-sepsis induced microvascular leak and downregulation of expression of *Angpt1* (*Ang1*), *Tek* (*Tie2*), and *Kdr* (*Vegfr2* or *Flk-1*) genes in the lung, kidney, and liver. These changes correlate with a decrease in RNA polymerase II density at these genes, and the greatest response was observed in the lung. ALI-sepsis reduced levels of transcription-permissive histone H3 lysine acetylation (H3KAc) at these loci in all examined tissues. Decreases in permissive H3K4m3 and H3Km2 marks were detected only in the lung. In contrast, only minimal alterations in transcription-repressive histone modifications (H3K27m3, H3K9m2, H3K9m3, and H4K20m3) were observed in all tissues.

**Conclusions:**

Our results demonstrate that decreases in transcription-permissive, but not increases in transcription-repressive, histone modifications at *Angpt1*, *Tek*, and *Kdr* are a systemic, rather than a lung-restricted, response, involving key end-organs in experimental ALI-sepsis. Given that ventilator-associated pneumonia is a major cause of sepsis in critically ill patients, elucidation of mechanisms mediating epigenetic alterations during sepsis provides fundamental new insights into the pathogenesis of sepsis-induced microvascular leak and subsequent end-organ injury/dysfunction.

**Electronic supplementary material:**

The online version of this article (doi:10.1186/s13054-015-0943-4) contains supplementary material, which is available to authorized users.

## Introduction

Sepsis is a common and devastating complication in critically ill patients and remains a major cause of morbidity and mortality [1]. Pneumonia and ventilator-associated pneumonia (VAP) represent leading causes of sepsis in intensive care units (ICUs) [2–5]. In large part, rapid progression to major end-organ (that is, lung, kidney, and liver) injury/dysfunction is responsible for the high morbidity and mortality of clinical sepsis. The last few decades of intense research and clinical trials have failed to improve clinical outcomes in sepsis management, and there is a pressing need to develop therapies of greater efficacy [5–9]. Historically, severe systemic inflammation—that is, release of tumor necrosis factor (TNF), monocyte chemoattractant protein-1 (MCP-1), interleukin-6 (IL-6), and other inflammatory mediators—was thought to play the major role in sepsis-induced multiple organ dysfunction syndrome (MODS) [10]. However, more recently, endothelial dysfunction and associated microvascular leak have emerged as critical pathogenic mechanisms in sepsis and MODS [9, 11, 12].

Tie receptors and their angiopoietin ligands (Tie2/Ang) and vascular endothelial growth factor receptors and their ligands (Vegfr/Vegf) are two endothelial receptor tyrosine kinase systems that play critical roles in the regulation of angiogenesis and endothelial function [13, 14]. Components of the Tie2/Ang and Vegfr/Vegf systems act, and are predominantly expressed, in the endothelium [13]. Although the role of abnormal Tie2/Ang signaling in mediating detrimental microvascular leak in sepsis and MODS is well established [15], recent studies have also implicated altered Vegfr/Vegf signaling in the pathogenesis of sepsis and MODS [16]. Sepsis-induced endothelial dysfunction driven by dysregulated Tie2/Ang and Vegfr/Vegf signaling reflects, in part, decreased levels of these angiogenic mediators and their receptors that, in turn, result from downregulation of these genes [15, 17]. Both Angpt1 and Angpt2 bind to the Tie2 receptor but often trigger opposite biological effects. Whereas Angpt1 can be protective, Angpt2 can worsen outcomes in sepsis, observations that are being used to develop biomarkers in sepsis [18–20]. Despite increasing appreciation of the role played by angiogenic genes in sepsis-induced MODS, the molecular mechanisms underlying their transcriptional regulation in this complex syndrome remain poorly understood.

Epigenetic processes play a critical role in transcriptional regulation by influencing genomic regulatory sequences in a cell type-specific and extracellular environment-dictated manner. Epigenetic language encompasses covalent histone modifications and DNA CpG methylation. Together these epigenetic modifications control chromatin accessibility to factors that regulate RNA polymerase II (Pol II) transcription (Additional file 1: Figure S1) [21, 22]. Although epigenetic regulation is one of the most intensely studied fields of biology today, relatively little is known about epigenetic regulation in sepsis and MODS. Identification of transcription factors and epigenetic modifiers engaged at injury-related genes could allow the development of small-molecule drugs to therapeutically target these factors in an effort to improve clinical outcomes in sepsis and MODS. In this regard, Nicodeme *et al*. showed that an epigenetic drug, bromodomain and extra terminal domain inhibitor (I-BET), decreased inflammatory responses and protected mice from lipopolysaccharide-induced sepsis and death [23].

Given the importance of pneumonia as a leading cause of sepsis in critically ill patients in the ICU, we set out to define transcriptional and epigenetic changes at genes encoding components of the Tie2/Ang and Vegfr/Vegf systems in the lung, kidney, and liver in an experimental mouse model of acute lung injury-induced sepsis (ALI-sepsis). We hypothesized that experimental ALI-sepsis (employing a previously validated murine model of VAP that combines *Staphylococcus aureus* lung infection and mechanical ventilation, resulting in lung injury and extra-pulmonary organ dysfunction [24]) would be associated with both decreased permissive and increased repressive epigenetic modifications at downregulated genes in the Tie2/Ang and Vegfr/Vegf systems in the lung. Contrary to our hypothesis, we found that ALI-sepsis was associated with only decreased permissive epigenetic modifications at these genetic loci in the lung. Moreover, these epigenetic changes were not confined to the lung but extended to the kidney and liver, two prominent extra-pulmonary end-organs affected in sepsis-related MODS [24, 25].

## Methods

### Reagents

Bovine serum albumin, phosphate-buffered saline (PBS), salmon sperm DNA, and protein A were from Sigma-Aldrich (St. Louis, MO, USA), and proteinase K was from Invitrogen (part of Thermo Fisher Scientific, Waltham, MA, USA). Ninety-six-well polypropylene plates for multiplex chromatin immunoprecipitation platform (Matrix ChIP) were from BioExpress (Kaysville, UT, USA). Formaldehyde, ethanol, NaCl, EDTA, Triton X-100, NP-40, Tris, leupeptin, PMSF, p-nitrophenyl phosphate, NaF, Na_3_VO_4_, Na_2_MoO_4_, and β-glycerophosphate were from Sigma-Aldrich. The antibodies were commercially available and are listed in Additional file 1: Table S2.

### Animal experiments

The specific protocol used in this study was approved by the Institutional Animal Care and Use Committee at the University of Washington. *S. aureus* was prepared and mice were inoculated and mechanically ventilated as previously described [25]. Briefly, for each experiment, a frozen aliquot of methicillin-sensitive *S. aureus* originally isolated from a patient with bacteremia was thawed and cultured on a sheep blood agar plate. The next morning, a single colony was selected and cultured overnight at 37 °C in tryptic soy broth. The morning after that, the bacteria were washed twice with saline and then resuspended in 2 mL of filtered, distilled water. Serial log dilutions were made and turbidity measured by OD540 (optical density at 540 nm). With a standard curve generated previously with OD540 versus bacterial concentration determined by quantitative culture, a working solution of approximately 2 × 10^8^ ± 10% was prepared with filtered, distilled water.

For each experiment, four mice were used. Two mice were anesthetized with 5% isoflurane while suspended by the front teeth at a 60° angle. The tongue was extruded with forceps, and 50 μL of *S. aureus* (approximately 10^7^ colony-forming units) was deposited in the oropharynx. After visual confirmation of aspiration, mice were placed on a nose cone with 5% isoflurane and intubated via tracheostomy with a 20-gauge blunt metal catheter. Intubated mice were connected to a MiniVent rodent ventilator (Harvard Biosciences, Hollison, MA, USA) and mechanically ventilated with a tidal volume of 10 mL/kg, a respiratory rate of 150 breaths per minute, a fraction of inspired oxygen (FiO_2_) of 0.21, and no end-expiratory pressure. Anesthesia was maintained with isoflurane (1.5%). Neuromuscular blockade was induced with pancuronium (0.02 mg in 0.2 mL subcutaneously). A second dose of pancuronium (0.01 mg in 0.1 mL) was given after 2 hours. Control mice were maintained in their cages and given an equivalent volume of PBS subcutaneously at time 0 and 2 hours.

After 6 hours, mice were deeply anesthetized with 5% isoflurane and euthanized by cardiac puncture and exsanguination. Lungs, kidneys, and livers were harvested, flash-frozen, and stored at −80 °C.

### Microvascular albumin leak measurements

Microscular leak was assessed by tissue albumin levels. Briefly, frozen tissues were homogenized in low detergent buffer (150 mM NaCl, 50 mM Tris, 5 mM EDTA, 0.005% NP-40, 0.01% Triton X-100, pH 7.5) by using a Bioruptor (Diagenode, Seraing, Belgium) (15 minutes, high power 30 seconds ON 30 seconds OFF, 4 °C). Homogenate was centrifuged (10,000 rpm, 5 minutes, 4 °C), and supernatant was boiled in SDS Laemmli buffer. Albumin levels were assessed by SDS-PAGE and Western blot analysis (anti-albumin SC-46293; Santa Cruz Biotechnology, Inc., Dallas, TX, USA; 1:2,000 dilution). Anti-β-actin antibody (A5441; Sigma-Aldrich; 1:5,000 dilution) was used as loading control. Albumin and β-actin band intensities were measured from scanned membrane images by using ImageJ64 software.

### RNA extraction and cDNA synthesis

RNA was extracted from frozen tissue fragments by using Trizol reagent in accordance with the protocol of the manufacturer. To synthesize cDNA, 400 ng of Trizol-extracted total RNA was reverse-transcribed with 200 units of MMLV reverse transcriptase (Invitrogen) and oligo-dT primers in 20-μL reactions. Reverse transcription (RT) reactions were diluted 100-fold with water prior to use in quantitative polymerase chain reaction (PCR) [26].

### Chromatin preparation and multiplex Matrix ChIP assay

The same protocol was used for all organs. The multiplex microplate Matrix ChIP method was previously described [27–29]. Briefly, for ChIP assays, frozen tissue fragments (10 to 20 mg) were cross-linked with formaldehyde, and chromatin was sheared by using a Bioruptor [27]. ChIP assays were done by using protein A-coated 96-well polypropylene microplates as described before [27]. Eluted DNA (1 to 2 μL) was used in 2-μL real-time PCRs (ABI7900HT). All PCRs were run in quadruplicates. PCR calibration curves were generated for each primer pair from a dilution series of total mouse genomic DNA. The PCR primer efficiency curve was fit to cycle threshold (Ct) versus log [genomic DNA concentration] by using an r^2^ best fit. DNA concentration values for each ChIP and input DNA sample were calculated from their respective average Ct values. Final results are expressed as fraction of input DNA [28]. Matrix ChIP PCR primers are shown in Additional file 1: Table S1, and a list of antibodies is shown in Additional file 1: Table S2.

### Statistical analysis and data display

GraphGrid Excel-based software tools were used to acquire, store, and analyze large data sets generated by the high-throughput Matrix ChIP platform [29]. Pair-wise *t* test was used to measure statistically significant differences, which are represented by the size of a circle for each comparison made: *P* <0.05 by a small circle, *P* <0.01 by a large circle, and non-significance by no circle [29].

## Results

### Serum albumin leak in the lung, kidney, and liver in an experimental ALI-sepsis model

**Fig. 1:**
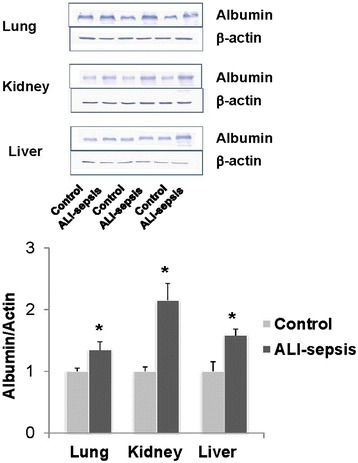
Increased albumin leak in the lung, kidney, and liver in an experimental acute lung injury-induced sepsis (ALI-sepsis) model. Combined *Staphylococcus aureus* pneumonia and mechanical ventilation in anesthetized mice was performed as previously described (Methods) [25]. Mice without any intervention served as controls. After 6 hours of treatment, the lungs, kidneys, and livers were harvested, flash-frozen, and stored at −80 °C. Western blot analysis of tissue albumin, top panel, was used to assess microvascular leak (Methods). Albumin and β-actin band intensities were measured from scanned membrane images by using ImageJ64 [54]. Albumin-to-β-actin ratios normalized to control tissues were calculated for each tissue; the graph depicts mean ± standard error of the mean (n = 3 mice in each group). *P <0.01 (*t* test) bottom panel

We previously developed a clinically relevant experimental murine model of ALI-sepsis in which mice with *S. aureus* pneumonia progress to sepsis-induced MODS when mechanically ventilated [25]. This model simulates a common clinical scenario in the ICU in which mechanically ventilated critically ill patients develop sepsis-induced MODS from a pulmonary source of infection. In this model, profound systemic release of multiple inflammatory mediators occurs within 6 hours, and it is not associated with extra-pulmonary dissemination of *S. aureus* [25]. These mediators or systemic release of pathogen-associated molecular pattern molecules (PAMPs) or endogenous danger-associated molecular pattern molecules (DAMPs/alarmins) or a combination of these could affect multiple organs. This ALI-sepsis model is characterized by increased serum creatinine and transaminases, indicating renal and hepatic impairment, respectively [25]. Endothelial dysfunction is another hallmark of sepsis [30, 31]. Serum albumin leak is often used to assess integrity of the microvascular barrier. Western blot analysis of proteins extracted from fragments of the lung, kidney, and liver showed higher tissue albumin levels (normalized to β-actin) in ALI-sepsis mice compared with controls (Fig. 1). The magnitude of observed differences is comparable to those reported for albumin leak in other experimental models of sepsis [32]. Thus, these tissue albumin measurements provide evidence for lung, kidney, and liver endothelial dysfunction and associated microvascular leak in this ALI-sepsis model.

### Experimental ALI-sepsis induced downregulation of angiogenic gene expression in the lung and extra-pulmonary organs

**Fig. 2:**
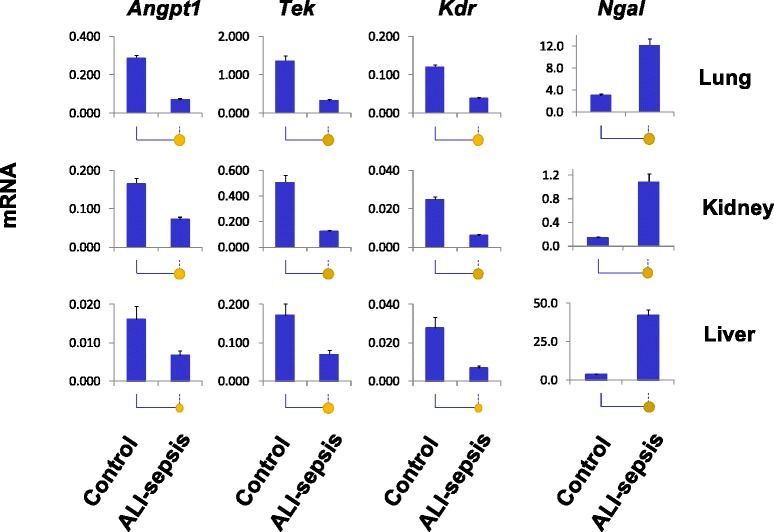
Analysis of changes in lung, kidney, and liver mRNA expression in experimental acute lung injury-induced sepsis (ALI-sepsis). Total RNA from ALI-sepsis mouse lungs, kidneys, and livers was used in reverse transcription reactions with oligo-dT primers. cDNA was used in real-time polymerase chain reaction with gene-specific primers (Additional file 1: Table S1). mRNA level of a given gene in each sample was normalized to β-actin transcript. Data are represented as mean ± standard error of the mean (n = 12 mice in each group). Statistical differences between two means (*P* value) are shown by the size of the solid circles: *P* <0.05 by a small circle, *P* <0.01 by a large circle, and non-significance by no circle

We examined changes in expression of several angiogenic genes in the lung, kidney, and liver. As shown in Fig. 2, mRNA levels of *Tek* receptor and its main cognate agonist/ligand, *Angpt1*, decreased in all three organs after 6 hours of experimental ALI-sepsis. These changes were most prominent in the lungs.

Vegfr2 is a homodimer receptor composed of Flk1 protein encoded by the *Kdr* gene [33]. Sepsis-induced downregulation of *Kdr* mRNA in the lung, kidney, and liver was similar in magnitude to the *Tek* mRNA changes (Fig. 2). In contrast to the marked downregulation of *Angpt1*, *Tek*, and *Kdr* expression in the lung, reductions of *Angpt2*, *Vegfα*, and *Flt1* (*Vegfr1*) mRNAs were small (Additional file 1: Figure S2). These genes were not further pursued in this study.

Neutrophil gelatinase-associated lipocalin (*Ngal*) (also known as *Lcn2*) is expressed in neutrophils and also in solid organs, where it is thought to provide protection against bacterial infection by sequestration of iron, thereby limiting bacterial growth [34]. *Ngal* has also gained much interest as a biomarker of acute kidney injury [35]. *Ngal* transcript levels increased in the lung, kidney, and liver in septic mice. We used upregulated *Ngal* expression as a pathophysiologically relevant control for transcriptional and epigenetic changes at downregulated angiogenic genes.

### Experimental ALI-sepsis decreased Pol II density at angiogenic genes

**Fig. 3:**
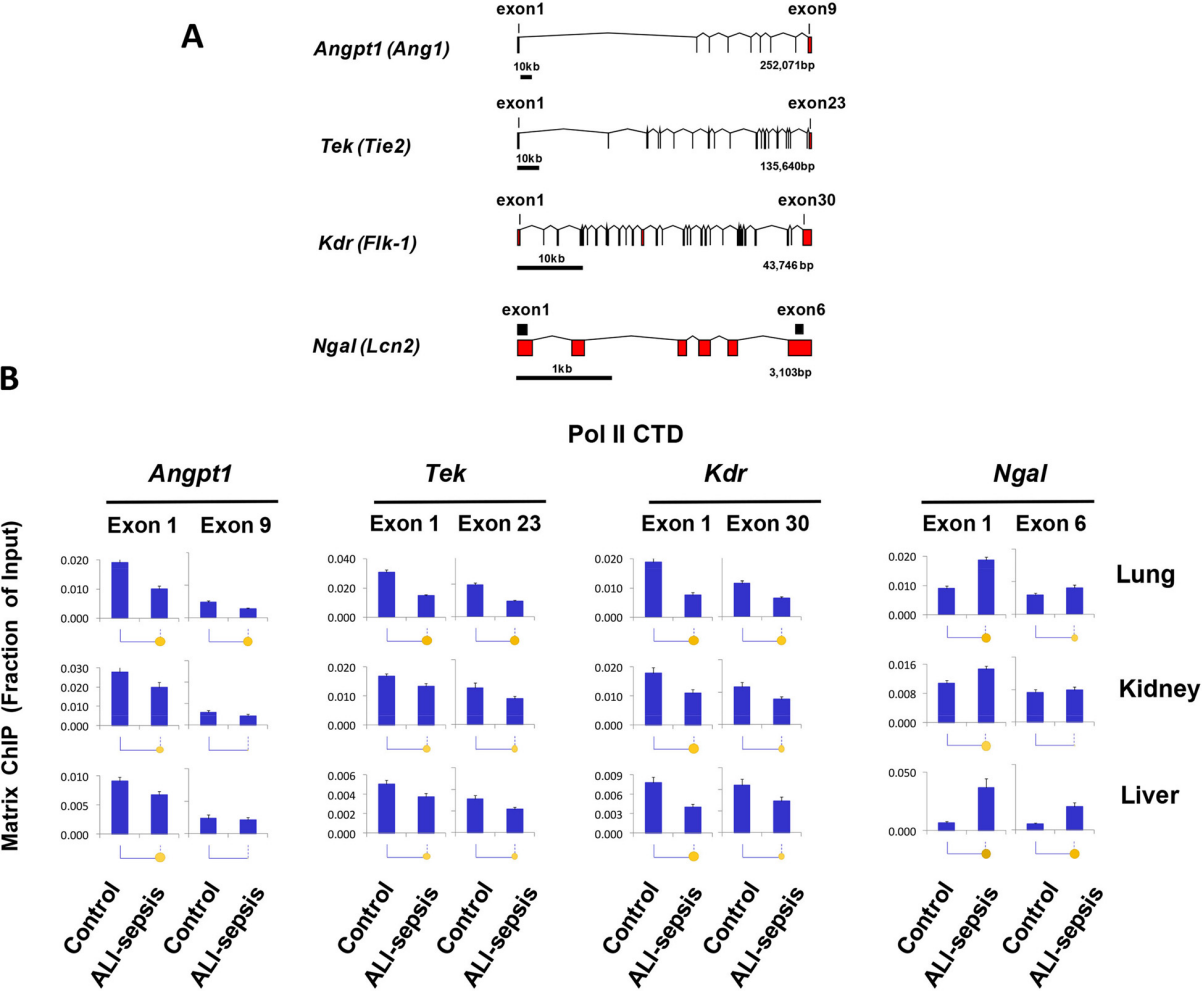
RNA polymerase II (Pol II) changes at *Angpt1*, *Tek*, *Kdr*, and *Ngal* genes in experimental acute lung injury-induced sepsis (ALI-sepsis). **(A)** Intron-exon organization of genes analyzed in these studies. Exons are shown as rectangles and introns as lines. Chromatin immunoprecipitation platform polymerase chain reaction (ChIP PCR) amplicons are shown as black boxes. **(B)** ALI was induced in anesthetized mice as in Fig. 1. Mice without any intervention served as controls. After 6 hours of treatment, the lungs, kidneys, and livers were harvested, flash-frozen, and stored at −80 °C. Sheared cross-linked chromatin was prepared from frozen lungs, kidneys, and livers. ChIP analysis was done by using an antibody to the Pol II C-terminal domain (CTD). ChIP DNA samples were analyzed by real-time quantitative PCR with primers to the first and last gene exons (A). Data represent mean ± standard error of the mean (n = 12 animals from each group), expressed as a fraction of input. Statistical differences are shown as in Fig. 2

Sepsis-induced changes in mRNA levels (Fig. 2) can result from changes in transcription or from changes in mRNA stability/degradation [29, 36]. To examine this issue, we used a monoclonal antibody to the Pol II C-terminal domain (CTD) in Matrix ChIP assays [28] to assess density of Pol II at angiogenic genes in the lungs, kidneys, and livers of septic mice. Matrix ChIP measurements were done at 5′ and 3′ ends of genes (Fig. 3A). After 6 hours of ALI-sepsis, Pol II levels at both the 5′ and 3′ ends of downregulated *Angpt1*, *Tek*, and *Kdr* genes were decreased in all three organs compared with control animals (Fig. 3B). ALI-sepsis-induced decreases were most pronounced in the lung. In contrast to the angiogenic genes, Pol II levels at the upregulated *Ngal* locus were higher in ALI-sepsis animals compared with controls (Fig. 3B). The magnitude of sepsis-induced Pol II changes at all the loci matched cognate mRNA changes (Fig. 2), suggesting that, at least in part, changes in mRNA levels reflected altered transcription of *Angpt1*, *Tek*, *Kdr*, and *Ngal* genes.

### Experimental ALI-sepsis decreased levels of transcription-permissive histone modifications at angiogenic genes

**Fig. 4:**
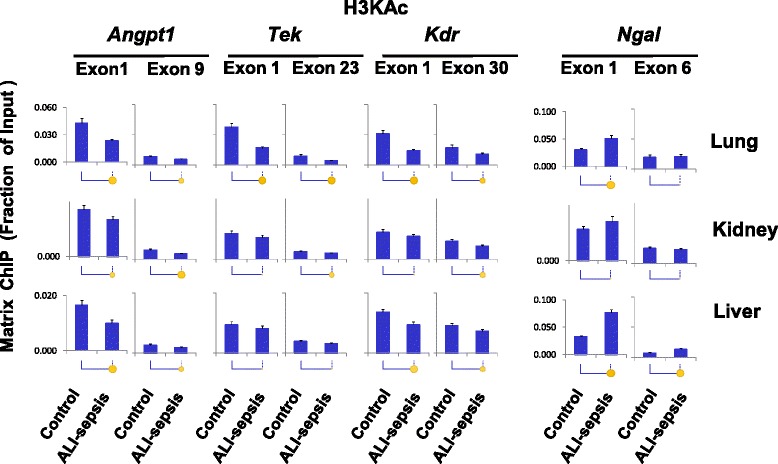
Matrix ChIP analysis of changes in histone H3 lysine acetylation (H3KAc) levels at *Angpt1*, *Tek*, *Kdr*, and *Ngal* genes in experimental acute lung injury-induced sepsis (ALI-sepsis). Sheared cross-linked lungs, kidneys, and livers were assayed by using an antibody to acetylated histone H3 lysine 9 and 14, H3KAc. ChIP analysis was done as in Fig. 3. Data represent mean ± standard error of the mean (n = 12 animals from each group), expressed as a fraction of input. Statistical differences between two means (*P* value) are shown as in Fig. 2. ChIP, chromatin immunoprecipitation platform

Compact chromatin structure is maintained, in part, by electrostatic interaction of positive histone amino acid residues and negatively charged DNA. Acetylation of histone lysine residues removes positive charges, reducing chromatin compaction and thereby permitting higher rates of Pol II transcription (Additional file 1: Figure S1) [37]. Histone acetyl-lysine modifications also serve as docking sites for factors that promote transcription via bromodomain interactions [38]. Histone lysine acetylation is higher at the 5′ compared with the 3′ ends of genes, suggesting their importance in transcription initiation and early steps of elongation [39]. Reduced transcription of angiogenic genes in organs of ALI-sepsis mice (Figs. 2 and 3) could conceivably be associated with deacetylation of histones. We used antibody against acetylated lysines 9 and 14 of histone H3 (H3KAc) in Matrix ChIP assay to assess changes in this transcriptionally permissive modification (Fig. 4). In the lungs of ALI-sepsis mice, H3KAc levels at 5′ ends of *Angpt1*, *Tek*, and *Kdr* were lower compared with control animals. Similar ALI-sepsis-induced histone H3 lysine deacetylation was observed in the kidney and the liver, but the decreases were smaller and at some sites not statistically significant. Total histone H3 levels at the genes remained unaltered or tended to be higher in organs of ALI-sepsis animals (Additional file 1: Figure S3), suggesting that reduced H3KAc levels did not reflect loss of nucleosomes. There was an increase in H3KAc at *Ngal* in all three organs, and the magnitude of change was greater in the lung and liver compared with the kidney. In summary, sepsis-induced H3KAc changes (Fig. 4) generally paralleled Pol II levels at the genes (Fig. 3), suggesting a functional link between ALI-sepsis-induced changes in histone lysine acetylation and rates of transcription.

**Fig. 5:**
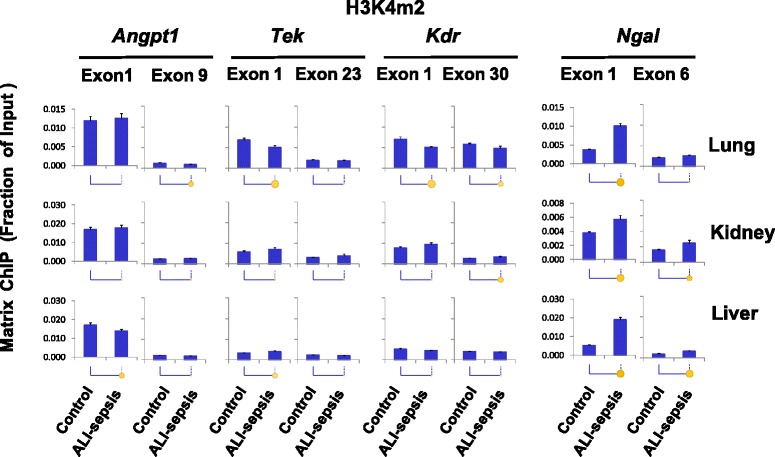
Changes in histone H3 lysine 4 di-methylation (H3K4m2) in experimental acute lung injury-induced sepsis (ALI-sepsis). Sheared cross-linked lung, kidney, and liver chromatin from mice were assayed by using an antibody to di-methylated histone H3 lysine 4, H3K4m2. Chromatin immunoprecipitation platform (ChIP) analysis was done as in Fig. 3. Data represent mean ± standard error of the mean (n = 12 animals from each group), expressed as a fraction of input. Statistical differences are shown as in Fig. 2

**Fig. 6:**
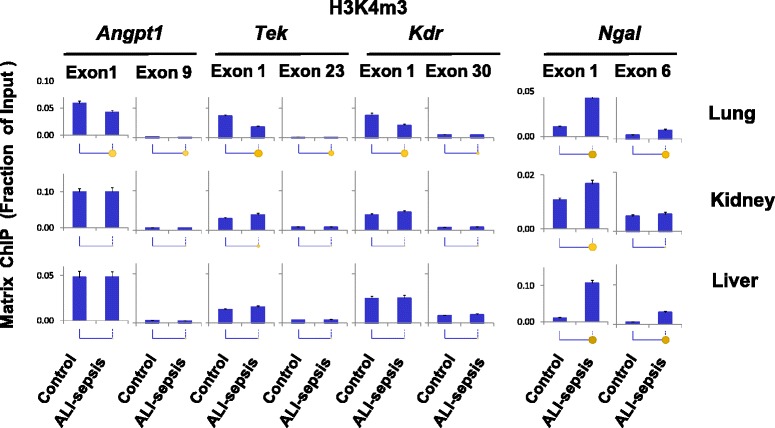
Changes in histone H3 lysine 4 tri-methylation (H3K4m3) in experimental acute lung injury-induced sepsis (ALI-sepsis). Sheared cross-linked lung, kidney, and liver chromatin from mice were assayed by using an antibody to H3K4m3. Chromatin immunoprecipitation platform (ChIP) analysis was done as in Fig. 3. Data represent mean ± standard error of the mean (n = 12 animals from each group), expressed as a fraction of input. Statistical differences are shown as in Fig. 2

Di- and tri-methylation of histone H3 lysine 4, H3K4m2 and H3K4m3, are other well-studied permissive chromatin modifications [21]. Histone lysine methylation provides docking sites for chromatin modifiers but, unlike acetylation, does not alter histone charge [37]. It has been suggested that H3K4m2 and H3K4m3 modifications open chromatin by recruitment of ATP-dependent chromatin remodelers [40]. These modifications are enriched at the 5′ ends of genes [41]. Consistently, for all of the angiogenic genes examined, H3K4m2 and H3K4m3 were higher at the 5′ compared with the 3′ ends in all organs (Figs. 5 and 6). Pol II decreases (Fig. 3) at *Tek* and *Kdr* genes in mice with ALI-sepsis were accompanied by decreases in H3K4m2 (Fig. 5) and H3K4m3 (Fig. 6) signals in the lung. In contrast, H3K4m2 and HK4m3 levels were largely unaltered by ALI-sepsis in the kidney and liver. Thus, ALI-sepsis-induced decreases in H3K4m2 and HK4m3 may account for the greater sepsis-induced repression of *Angp1*, *Tek*, and *Kdr* transcription in the lung compared with extra-pulmonary organs (Fig. 3).

These results suggest organ-specific differences in epigenetic responses to ALI-sepsis. Some of the differences may reflect the magnitude of local lung versus remote responses to ALI-sepsis. Consistent with elevated transcription, ALI-sepsis-induced increases in H3K4m2 and H3K4m3 levels were observed at *Ngal* in the lung, kidney, and liver. The magnitude of H3K4m2 (Fig. 5) and H3K4m3 (Fig. 6) changes at *Ngal* correlated well with the observed ALI-sepsis-induced increase in Pol II levels at this locus in the different organs (Fig. 3). Furthermore, the magnitude of ALI-sepsis-induced H3K4m2 and H3Km3 increases at *Ngal* in the different organs correlated with changes in H3 acetylation levels at this locus. These observations imply that these two permissive epigenetic modifications act in concert to generate robust *Ngal* transcriptional responses during ALI-sepsis.

Results of the above experiments imply that ALI-sepsis-induced decreases in both permissive histone lysine acetylation and methylation play a role in aberrant transcription of angiogenic genes in the lung. In contrast, alterations in histone lysine acetylation appear more important than alterations in histone methylation in aberrant transcription of angiogenic genes in extra-pulmonary organs.

### Experimental ALI-sepsis induced minimal transcription-repressive histone modifications at angiogenic genes

**Fig. 7:**
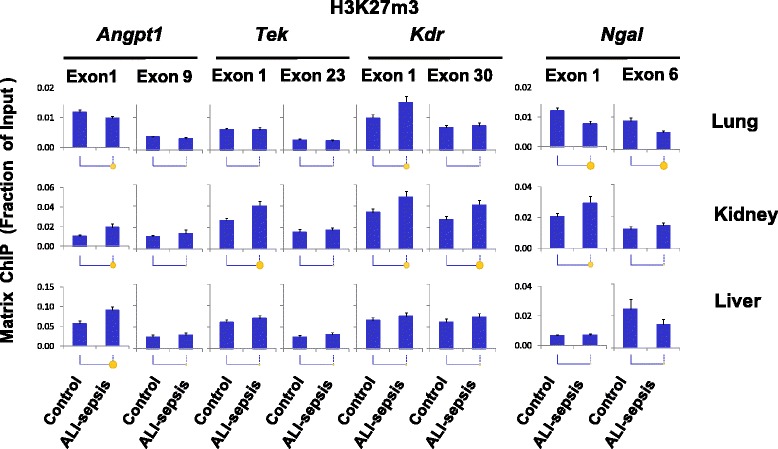
Changes in histone H3 lysine 27 tri-methylation (H3K27m3) in experimental acute lung injury-induced sepsis (ALI-sepsis). Sheared cross-linked lung, kidney, and liver chromatin from mice were assayed by using an antibody to H3K27m3. Chromatin immunoprecipitation platform (ChIP) analysis was done as in Fig. 3. Data represent mean ± standard error of the mean (n = 12 animals from each group), expressed as a fraction of input. Statistical differences between are shown as in Fig. 2

We examined several histone modifications (H3K27m3, H3K9m2, H3K9m3, and H4K20m3) associated with repressed genes (Fig. 7 and Additional file 1: Figures S4-S6). These modifications downregulate gene expression by increasing chromatin compaction, thereby decreasing access of transcription factors and impeding Pol II recruitment [21, 37, 42]. In the ALI lung, there was an increase in H3K27m3 levels at the 5′ end of *Kdr* but not at other angiogenic loci. In the kidney, ALI-sepsis was associated with small increases in H3K27m3 levels at *Angpt1*, *Tek*, and *Kdr*, but in the liver the increase was detected at the *Angpt1* gene. At the upregulated *Ngal* locus, H3K27m3 loss was detected only in the lung.

H3K9m2 and H3K9m3 are well-studied repressive marks that are enriched in compacted heterochromatin [42, 43]. There were no consistent sepsis-induced changes in H3K9m2/3 levels at the angiogenic genes (Additional file 1: Figures S4 and S5), suggesting that these epigenetic marks did not contribute to *Angp1*, *Tie2*, and *Kdr* repression in the organs examined. ALI-sepsis caused H3K9m2 and H3K9m3 de-methylation, albeit at low levels, at *Ngal* in the lung and kidney, and this could potentially contribute to increased *Ngal* transcription in these organs.

Histone H4 lysine 20 tri-methylation, H4K20m3, is another repressive mark enriched in compacted chromatin [21, 44]. Matrix ChIP analysis showed no consistent sepsis-induced changes in H4K20m3 at angiogenic and *Ngal* genes in any of the organs (Additional file 1: Figure S6).

Taken together, these results suggest that changes in repressive epigenetic modifications play an insignificant role in ALI-sepsis-induced downregulation of pulmonary and extra-pulmonary *Angp1*, *Tie2*, and *Kdr*. However, decreased repressive histone modifications may contribute to sepsis-induced *Ngal* expression in some organs (Fig. 7 and Additional file 1: Figures S4-S6).

## Discussion

Sepsis is characterized by downregulation of *Angpt1*, *Tek*, *Kdr*, and other angiogenic genes. These alterations have been implicated in the pathogenesis of sepsis-induced microvascular leak and end-organ injury/dysfunction [17, 45, 46]. Here, we present evidence that dysregulation of *Angpt1*, *Tek*, and *Kdr* caused by experimental ALI-sepsis is associated with a reduction in levels of Pol II at these loci in the lung, kidney, and liver, thereby providing, for the first time, evidence for the systemic transcriptional repression of these angiogenic genes in sepsis. This finding prompted our hypothesis that ALI-sepsis would be associated with both decreased permissive and increased repressive epigenetic modifications at *Angpt1*, *Tek*, and *Kdr.*

To explore the epigenetic basis of sepsis-induced transcriptional dysregulation of angiogenic genes, we compared several important permissive and repressive histone modifications with Pol II levels along these genes. There was positive correlation between ALI-sepsis-induced reduction of Pol II levels (Fig. 3) at *Angpt1*, *Tek*, and *Kdr* and deacetylation of H3KAc at these loci in the lung, kidney, and liver (Fig. 4). There was also concordance between ALI-sepsis-induced increases in Pol II and H3KAc levels at the upregulated *Ngal* locus in these organs. Given that histone lysine acetylation at gene promoters is a well-established transcription-permissive epigenetic modification [37], this analysis suggests that H3KAc changes play a mechanistic role in sepsis-induced dysregulation of angiogenic genes in the lung, kidney, and liver. The observed ALI-sepsis-induced Pol II decline (Fig. 3) also correlated with loss of H3K4m2 (Fig. 5) and H3K4m3 (Fig. 6) at angiogenic genes in the lung. Changes in these modifications at angiogenic genes in the other organs were minimal. Thus, although loss of these permissive modifications in the lung could play a role in sepsis-induced downregulation of pulmonary angionenic genes, their relative contribution to transcriptional dysregulation in extra-pulmonary end-organs in this model of ALI-sepsis appears minimal.

In contrast to the permissive epigenetic changes described above, experimental ALI-sepsis induced relatively few changes in repressive epigenetic modifications at *Angpt1*, *Tek*, and *Kdr* in the lung and extra-pulmonary end-organs. Except for changes in H3K27m3 levels in septic organs (Fig. 7), analysis of repressive post-translational modifications H3K9m2, H3K9m3, and H4K20m3 (Additional file 1: Figures S4-S6) revealed no significant ALI-sepsis-induced changes at the angiogenic genes in the lung, kidney, and liver. ALI-sepsis-induced H3K27m3 alterations were generally minimal and present primarily in the kidney as opposed to the other organs. Thus, alteration in repressive histone modifications may not play a major role in dysregulation of angiogenic genes in this model of ALI-sepsis and does not appear to be an epigenetic hallmark of sepsis, at least at the examined time point, contrary to our initial hypothesis.

There are several limitations in the present study. Given the remaining pre-analytical technical challenges in preparing a large number of samples for epigenetic analysis, we assessed only one time point of ALI-induced sepsis and have focused on *Angpt1*, *Tek*, and *Kdr*, which exhibited the most pronounced mRNA downregulation. At the time point examined, changes in *Angpt2*, *Vagfα*, and *Flt1* expression were small (Additional file 1: Figure S2). Sepsis-induced alteration in angiogenic gene expression can be time-dependent and therefore it is possible that either downregulation [45] or upregulation [46] of *Angpt2*, *Vagfα*, and *Flt1* was more robust at other time points. To evaluate whether mechanical ventilation has an independent effect on genes of interest, we analyzed microarray data from our previously published study that included normal mice and mice subjected to mechanical ventilation for 6 hours with the same protocol used in the present study, including anesthesia and paralysis [47]. Importantly, mechanical ventilation alone had a very modest effect on the expression of *Tek*, *Kdr*, *Angpt1*, *Angpt2*, or *Ngal* compared with control, unventilated mice (data not shown). Given the small changes in mRNA expression, we did not carry out epigenetic experiments with a mechanical ventilation-only group in the present study. It is anticipated that novel pre-analytical approaches and further advancement in epigenetic platforms as well as single-cell analysis will enable transcriptional and epigenetic studies of genes whose expression is not as markedly altered in whole tissue samples. Sepsis-induced reductions of angiogenic protein levels have previously been reported in the lung [46, 48, 49], kidney [50, 51], and liver [46]. Similar observations were made for sepsis downregulation of Kdr protein in the lung [45, 52]. Translation or protein degradation or both could be other important layers controlling expression of angiogenic genes in sepsis.

**Fig. 8:**
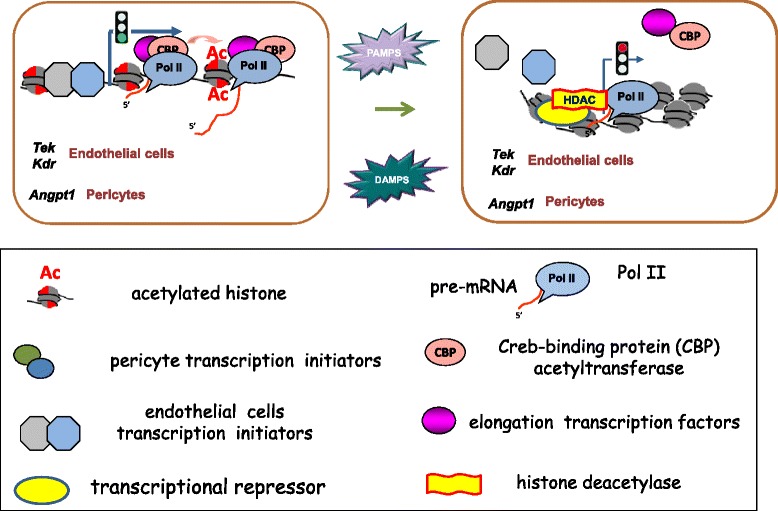
Proposed model for sepsis-induced downregulated transcription of angiogenic genes in endothelial cells and pericytes. Constitutively, epigenetic modifiers, including those that maintain permissive histone acetylation, are bound to genes by transcription factors and polymerase II (Pol II) machinery. During sepsis, systemic release of pathogen-associated molecular pattern molecules (PAMPs) or endogenous danger-associated molecular pattern molecules (DAMPs) or both initiates signal pathways that reduce activity of gene-bound transcription initiation or elongation factors or both. These transcription factor changes alter the balance of modifiers, shifting open chromatin toward more compact structures and contributing to downregulation of Pol II transcription of *Tek* and *Kdr* in endothelial cells and *Angpt1* in pericytes

The level of any given histone modification reflects the balance between epigenetic “writers” and “erasers” [21]. *Tek* and *Kdr* are expressed predominantly in endothelial cells, whereas *Angpt1* is expressed primarily in pericytes [17]. Considering the analysis performed in this study, we propose a model of sepsis-induced epigenetic modifications of angiogenic genetic loci at major target end-organs as depicted in Fig. 8. In contrast to ALI-sepsis-induced multi-organ increase in *Ngal* (Fig. 2) as well as nuclear factor-kappa-B (NF-κB)-responsive genes, including *Tnf*, *Mcp-1*, *IL-6*, and *Cox2* (Additional file 1: Figure S7), the angiogenic gene expression decreased in the lung, kidney, and liver. Selective downregulation of these angiogenic genes may reflect ALI-sepsis effects on endothelial cell-specific transcription factors that control Pol II recruitment and epigenetic modifiers. Constitutively, the epigenetic balance is shifted toward higher levels of permissive epigenetic marks at angiogenic loci in both endothelial cells and pericytes by the activity of cell type-specific and general transcription factors. During sepsis, systemic release of PAMPs [42] or DAMPs [21, 29] or both initiates signal pathways that reduce levels of activating transcription factors at these angiogenic loci (Fig. 8). As a result, there is a reduction of permissive histone modifications at *Tek* and *Kdr* in endothelial cells and *Angpt1* in pericytes, resulting in repression of Pol II transcription in both cell types (Fig. 8). Among the histone modifications examined, lysine acetylation was the most consistent sepsis-responsive epigenetic modification (Fig. 4). It is conceivable that transcription factors that control histone lysine acetyltransferases (HATs) and deacetylases (HDACs) are more sensitive to mediators of sepsis but that repressive histone lysine modifiers are relatively resistant. With increasing availability of small molecules that target different classes of chromatin modifiers, identification of epigenetic enzymes responsive to septic signals will be an important translational goal to elucidate key molecular mechanisms underlying the pathogenesis of clinical sepsis.

## Conclusions

In summary, sepsis remains a major cause of morbidity and mortality in critically ill patients, and VAP is an important cause of sepsis in the ICU. Specific therapeutic options beyond supportive care and antimicrobial agents for pathogen eradication currently do not exist for patients with ALI-induced sepsis. With the increasing awareness that the endothelium plays a major role in end-organ responses to sepsis, we applied the multiplex Matrix ChIP platform to identify systemic transcriptional and epigenetic changes associated with sepsis-mediated downregulation of angiogenic genes in target organs. This analysis revealed important changes in permissive epigenetic histone modifications at *Angpt1*, *Tek*, and *Kdr* in the lung, kidney, and liver. However, contrary to our initial hypothesis, ALI-induced changes in repressive epigenetic histone modifications at these genetic loci were relatively minor. This approach and our findings open up a fundamentally new venue to define the epigenetic basis of sepsis-induced microvascular leak and end-organ injury/dysfunction while also identifying the epigenetic modifiers underlying these alterations. Our data suggest that transcriptionally mediated alterations in *Angpt1*, *Tek*, and *Kdr* during sepsis may potentially be modulated through targeting histone acetylases/deacetylases. Several drugs that target this pathway are currently in clinical testing for other diseases [53] but have not been evaluated in sepsis. Mapping epigenetic modifiers tethered to angiogenic loci during sepsis is a promising approach to discover novel therapeutic targets for innovative drug development to mitigate pulmonary injury and extra-pulmonary organ injury in ALI and sepsis.

## Key messages

- Experimental ALI downregulates angiogenic gene expression not only in the lung but also in extra-pulmonary organs.
- Experimental ALI-induced downregulation of angiogenic genes in all examined organs is transcriptionally mediated.
- Experimental ALI-induced downregulation of angiogenic genes is associated with decreased permissive but minimal changes in repressive histone modifications.
- Identification of histone deacetylation at downregulated angiogenic genes as a systemic response to sepsis opens up new possibilities of using rational-design epigenetic therapy (for example, histone deacetylase inhibitor) to mitigate sepsis-associated microvascular dysfunction and endothelial leak.

## Additional file

Additional file 1: Table S1. Polymerase chain reaction (PCR) primers. **Table S2.** Chromatin immunoprecipitation platform (ChIP) antibodies. **Figure S1.** Example of epigenetic regulation of chromatin structure and transcription. **Figure S2.** Analysis of changes in lung *Angpt2*, *Vegfα*, and *Flt1* mRNA expression in experimental acute lung injury-induced sepsis (ALI-sepsis). **Figure S3.** Multiplex chromatin immunoprecipitation platform (Matrix ChIP) analysis of histone H3 density at *Angpt1*, *Tek*, *Kdr*, and *Ngal* in experimental acute lung injury-induced sepsis (ALI-sepsis). **Figure S4.** Multiplex chromatin immunoprecipitation platform (Matrix ChIP) analysis of changes in repressive histone H3 lysine 9 di-methylation (H3K9m2) at *Angpt1*, *Tek*, *Kdr*, and *Ngal* genes in experimental acute lung injury-induced sepsis (ALI-sepsis). **Figure S5.** Multiplex chromatin immunoprecipitation platform (Matrix ChIP) analysis of changes in repressive histone H3 lysine 9 tri-methylation (H3K9m3) in experimental acute lung injury-induced sepsis (ALI-sepsis). **Figure S6.** Multiplex chromatin immunoprecipitation platform (Matrix ChIP) analysis of changes in histone H4 lysine 20 tri-methylation (H4K20m3) in experimental acute lung injury-induced sepsis (ALI-sepsis). **Figure S7.** Analysis of changes in the lung, kidney, and liver of *Tnf*, *Mpc-1*, *IL-6*, and *Cox2* mRNA expression in experimental acute lung injury-induced sepsis (ALI-sepsis).

## Abbreviations

ALI-sepsis: acute lung injury-induced sepsis
Ang: angiopoietin
ChIP: chromatin immunoprecipitation platform
Ct: cycle threshold
DAMP: danger-associated molecular pattern molecule
ICU: intensive care unit
IL-6: interleukin-6
Matrix ChIP: multiplex chromatin immunoprecipitation platform
MCP-1: monocyte chemoattractant protein-1
MODS: multiple organ dysfunction syndrome
OD540: optical density at 540 nm
PAMP: pathogen-associated molecular pattern molecule
PBS: phosphate-buffered saline
PCR: polymerase chain reaction
Pol II: polymerase II
TNF: tumor necrosis factor
VAP: ventilator-associated pneumonia
VEGF: vascular endothelial growth factor
VEGFR: vascular endothelial growth factor receptor
