# Supplementary material for: Experimental acute lung injury induces multi-organ epigenetic modifications in key angiogenic genes implicated in sepsis-associated endothelial dysfunction
Source: Crit Care. 2015 May 11;19(1):225. doi: 10.1186/s13054-015-0943-4 (PMC4449602; doi:10.1186/s13054-015-0943-4)
Supplement: Additional file 1: Table S1. — Polymerase chain reaction (PCR) primers. Table S2. Chromatin immunoprecipitation platform (ChIP) antibodies. Figure S1. Example of epigenetic regulation of chromatin structure and transcription. Figure S2. Analysis of changes in lung Angpt2, Vegfα, and Flt1 mRNA expression in experimental acute lung injury-induced sepsis (ALI-sepsis). Figure S3. Multiplex chromatin immunoprecipitation platform (Matrix ChIP) analysis of histone H3 density at Angpt1, Tek, Kdr, and Ngal in experimental acute lung injury-induced sepsis (ALI-sepsis). Figure S4. Multiplex chromatin immunoprecipitation platform (Matrix ChIP) analysis of changes in repressive histone H3 lysine 9 di-methylation (H3K9m2) at Angpt1, Tek, Kdr, and Ngal genes in experimental acute lung injury-induced sepsis (ALI-sepsis). Figure S5. Multiplex chromatin immunoprecipitation platform (Matrix ChIP) analysis of changes in repressive histone H3 lysine 9 tri-methylation (H3K9m3) in experimental acute lung injury-induced sepsis (ALI-sepsis). Figure S6. Multiplex chromatin immunoprecipitation platform (Matrix ChIP) analysis of changes in histone H4 lysine 20 tri-methylation (H4K20m3) in experimental acute lung injury-induced sepsis (ALI-sepsis). Figure S7. Analysis of changes in the lung, kidney, and liver of Tnf, Mpc-1, IL-6, and Cox2 mRNA expression in experimental acute lung injury-induced sepsis (ALI-sepsis). [file 13054_2015_943_MOESM1_ESM.pdf]

Critical Care: 14940701461562361494070146156236R **Supplementary material**

Experimental acute lung injury induces multi-organ epigenetic modifications in key angiogenic genes implicated in sepsis-associated endothelial dysfunction

.[1,2]Karol Bomsztyk, [1]Daniel Mar, [1]Dowon An, [1]Roya Sharifian, [1]Michal Mikula, [1,2,3]Sina A. Gharib, [1,2,3]William A. Altemeier, [1,2,3]W. Conrad Liles and [1,2]Oleg Denisenko

[1]UW Medicine South Lake Union, University of Washington, Seattle, WA 98109, USA;

[2]Department of Medicine, University of Washington, Seattle, WA 98195, USA.

[3]Center for Lung Biology, University of Washington, Seattle, WA 98109, USA

Karol Bomsztyk <karolb@u.washington.edu>,

Daniel Mar <danmar@uw.edu>,

Dowon An <dowon@u.washington.edu>,

Roya Sharifian <roya_sharifian@yahoo.com>,

Michal Mikula <mikula.michal@gmail.com>,

Sina A. Gharib <sagharib@u.washington.edu>,

William A. Altemeier" <billa@u.washington.edu>,

W Conrad Liles <wcliles@uw.edu>,

Oleg Denisenko <odenis@u.washington.edu>,

Address for correspondence:

Karol Bomsztyk

UW Medicine at Lake Union , Box 358050

Seattle, WA 98109 USA

Tel (206) 616-7949

Table S1. PCR primers

Primer	Sequence (all mouse)
Tek (Tie2) Exon1 FWD	GTTGTTGAAAGCTTCCCAGG
Tek (Tie2) Exon1 REV	ACTAAGCCGGCTAAAGAGTC
Tek (Tie2) Exon23 FWD	AGGAAGAAAAGCGAGGGAAA
Tek (Tie2) Exon123 REV	CCCTCTCTTCTGCTACTTGG
Angpt1 (Ang1)Exon1 FWD	CAGCGAAGAATGCAAAGGAA
Angpt1 (Ang1) Exon1 REV	GAGAAGAGCAAGCTTTGCAG
Angpt1 (Ang1) Exon9 FWD	AGTTGGAACAGCCCATTGTA
Angpt1 (Ang1) Exon9 REV	TGAAGGCCTACGAACACTTT
Kdr (Flk-1) Exon1 FWD	GAGTCTGTGCCTGAGAACTG
Kdr (Flk-1) Exon1 REV	CAGAACCACAGAGCGACA
Kdr (Flk-1) Exon30 FWD	CGC TCACCTCCTGTTTAAATG
Kdr (Flk-1) Exon30 REV	CACCTCTCTCGTGATTTCCA
Ngal (Lcn2) Exon1 FWD	AGTTCTGAGTTGAGTCCTGG
Ngal (Lcn2) Exon1 REV	CCTAGTAGCTGTGGAAACCA
Ngal (Lcn2) Exon6 FWD	AGATGCTCCTTGGTATGGTG
Ngal (Lcn2) Exon6 REV	CTGTCTGCCACTCCATCTTT

Table S2. ChIP-antibodies

Figure	Antibody	Catalog No	Source	Manufacturer
3	Pol II CTD (4H8)	sc-47701	Mouse monoclonal	Santa Cruz
4	H3K9/14Ac	06-599	Rabbit polyclonal	Millipore
S3	H3	06-755	Rabbit polyclonal	Millipore
5	H3K4m2	07-030	Rabbit polyclonal	Millipore
6	H3K4m3	MA511199	Mouse monoclonal	Pierce
7	H3K27m3	ab6002	Mouse monoclonal	ABCAM
S4	H3K9m2	07-212	Rabbit polyclonal	Millipore
S5	H3K9m3	PA527042	Rabbit polyclonal	Pierce
S6	H4K20m3	07-463	Rabbit polyclonal	Millipore

Supplementary Figures

Fig.S1

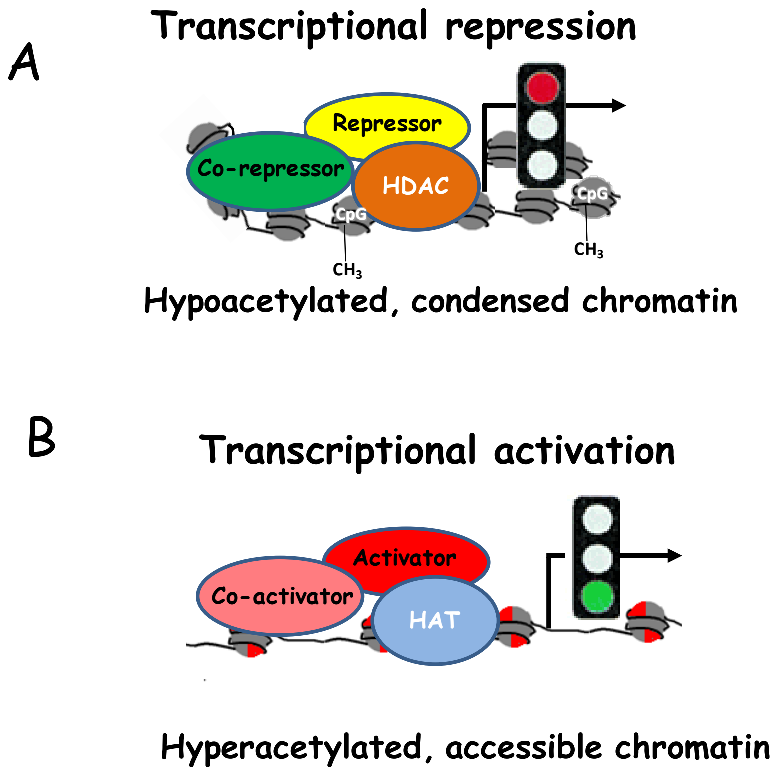

Fig.S1. Example of epigenetic regulation of chromatin structure and transcription.

A. Compact chromatin is an obstacle to transcription. Condensely packed nucleosomes (gray) encompassing repressed genes have hypoacetylated histone lysine residues and contain hypermethylated DNA CpGs (CpG-CH_3). Hypoacetylation is maintained by histone deacetylases (HDAC) recruited to repressed loci by transcriptional repressors. **B.** Transcriptional activators recruit histone acetyltransferases (HATs) resulting in increased histone lysine acetylation (red segments in nucleosomes), a modification that removes positive charges from nucleosomes lessening the electrostatic interaction with negatively changed DNA, loosening up chromatin structure providing increased access to Pol II machinery.

Fig.S2

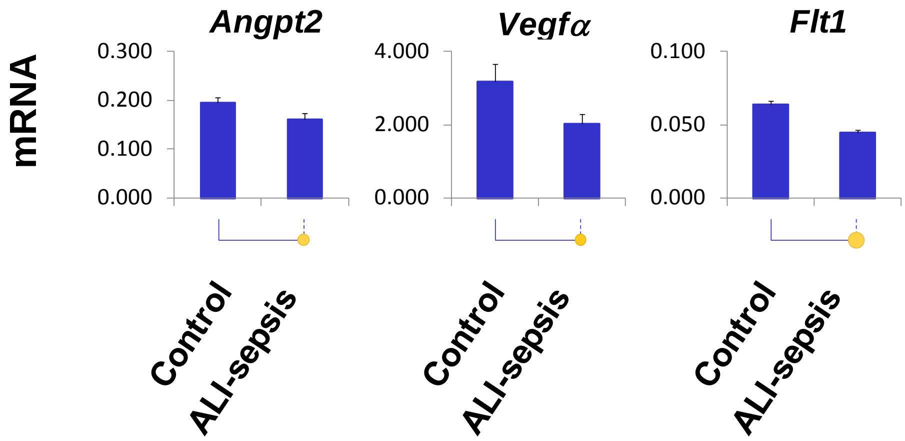

Fig.S2. Analysis of changes in lung *Angpt2*, *Vegfα* and *Flt1* mRNA expression in experimental ALI-sepsis. cDNA was used in real time PCR with gene specific primers. mRNA level of a given gene in each sample was normalized to β–actin transcript. Data are represented as mean ± SEM, n= 12 mice in each group. Statistical differences between two means (p value) are shown by the size of the solid circles: $p<0.05$ for small circle, $p<0.01$ for large circle, and no circle indicating the differences are not statistically significant.

Fig.S3

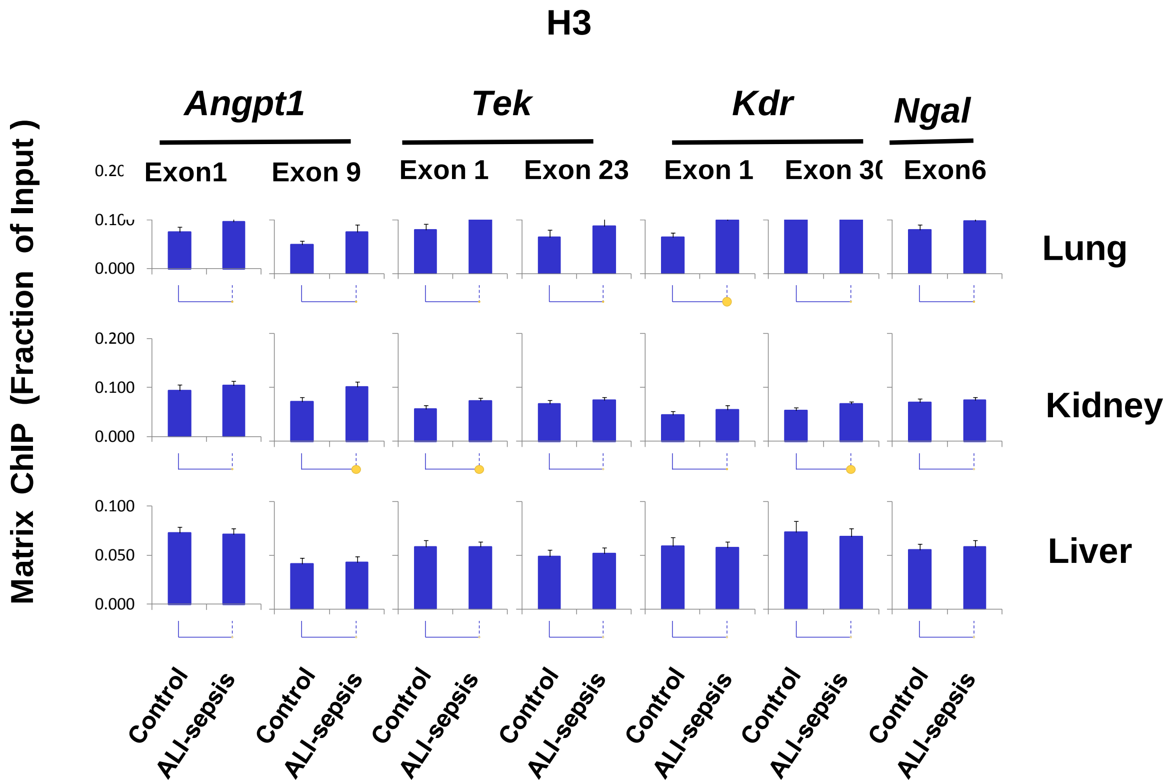

Fig.S3. Matrix ChIP analysis of histone H3 density at *Angpt1*, *Tek*, *Kdr* and *Ngal* in experimental ALI-sepsis. Sheared cross-linked chromatin from mice lungs, kidney were assayed using an antibody total histone H3. ChIP analysis was done as in Fig.3. Data represent mean ± SEM (n=12 animals from each group), expressed as a fraction of input.

Fig.S4

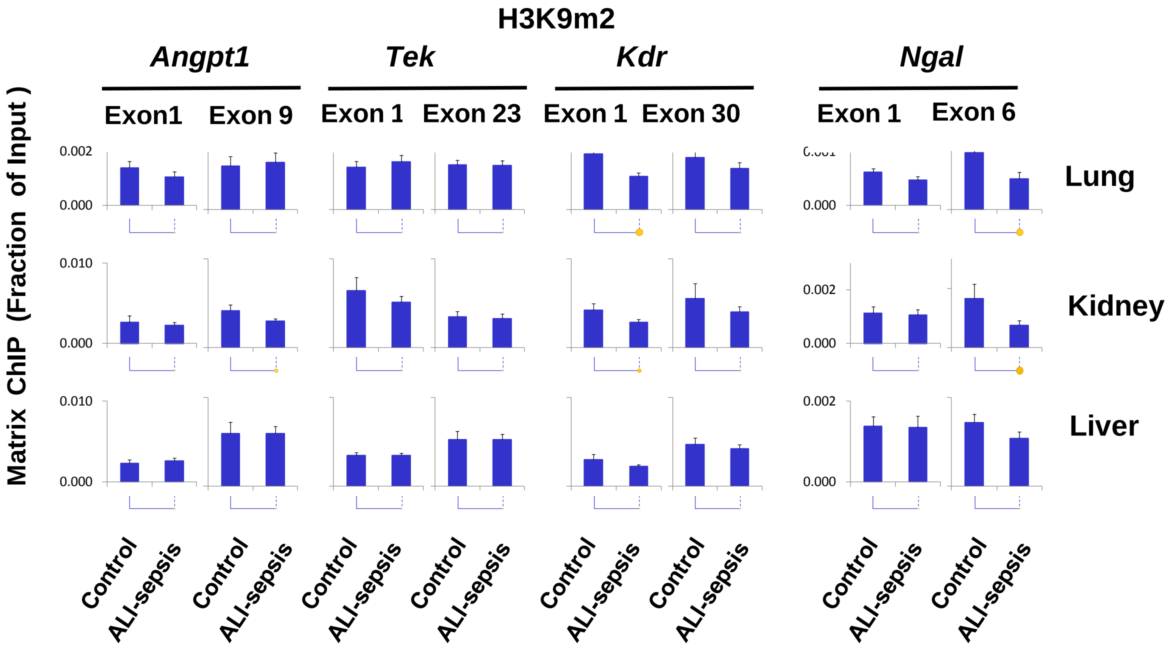

Fig.S4. Matrix ChIP analysis of changes in repressive histone H3 lysine 9 dimethylation (H3K9m2) at *Angpt1, Tek, Kdr* and *Ngal* genes in experimental ALI-sepsis. Sheared cross-linked lungs, kidneys and livers chromatin from mice were assayed using an antibody to H3K9m2. ChIP analysis was done as in Fig.3. Data represent mean ± SEM (n=12 animals from each group), expressed as a fraction of input.

Fig.S5

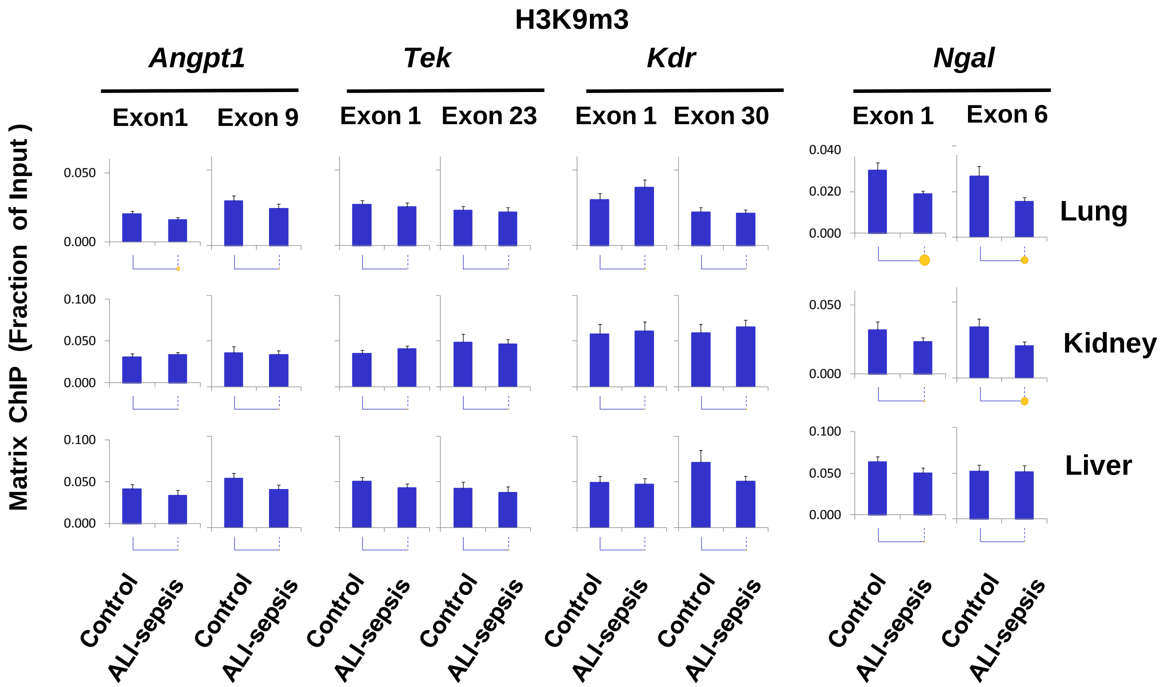

Fig.S5. Matrix ChIP analysis of changes in repressive histone H3 lysine 9 tri-methylation (H3K9m3) in experimental ALI-sepsis.. Sheared cross-linked lungs, kidneys and livers chromatin from mice were assayed using an antibody to H3K9m3. ChIP analysis was done as in Fig.3. Data represent mean ± SEM (n=12 animals from each group), expressed as a fraction of input.

Fig.S6

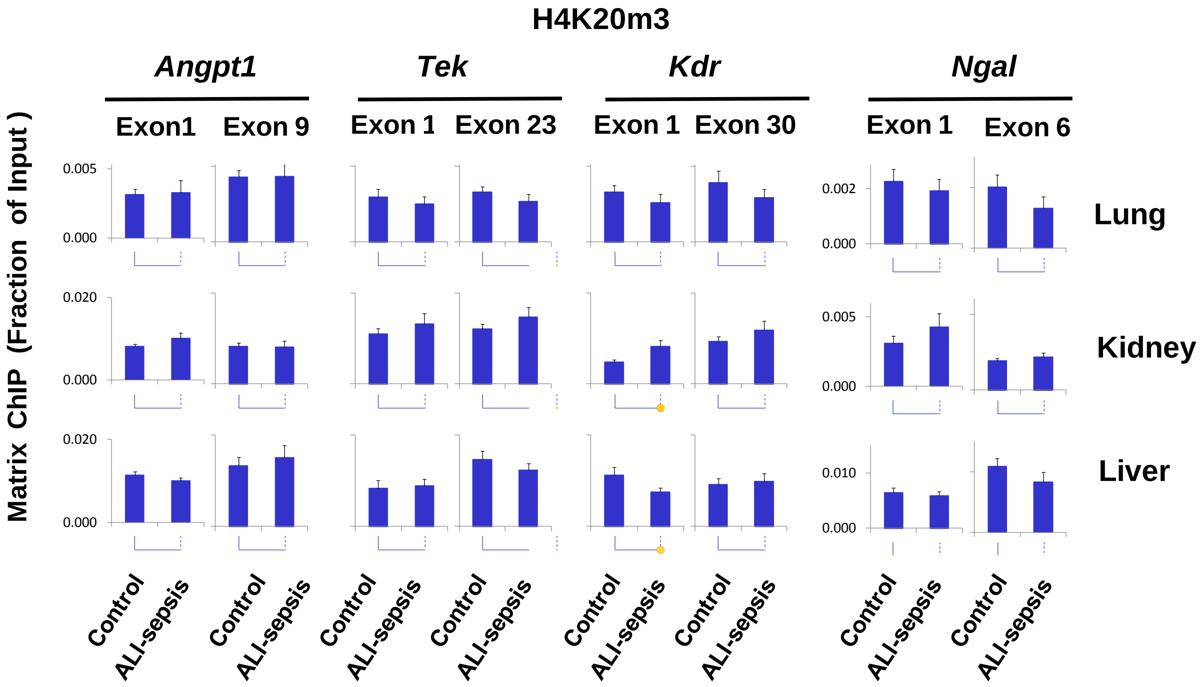

Fig.S6. Matrix ChIP analysis of changes in histone H4 lysine 20 tri-methylation (H4K20m3) in experimental ALI-sepsis. Sheared cross-linked lungs, kidneys and livers chromatin from mice were assayed using an antibody to H3K27m3. ChIP analysis was done as in Fig.3. Data represent mean ± SEM (n=12 animals from each group), expressed as a fraction of input.

Fig.S7

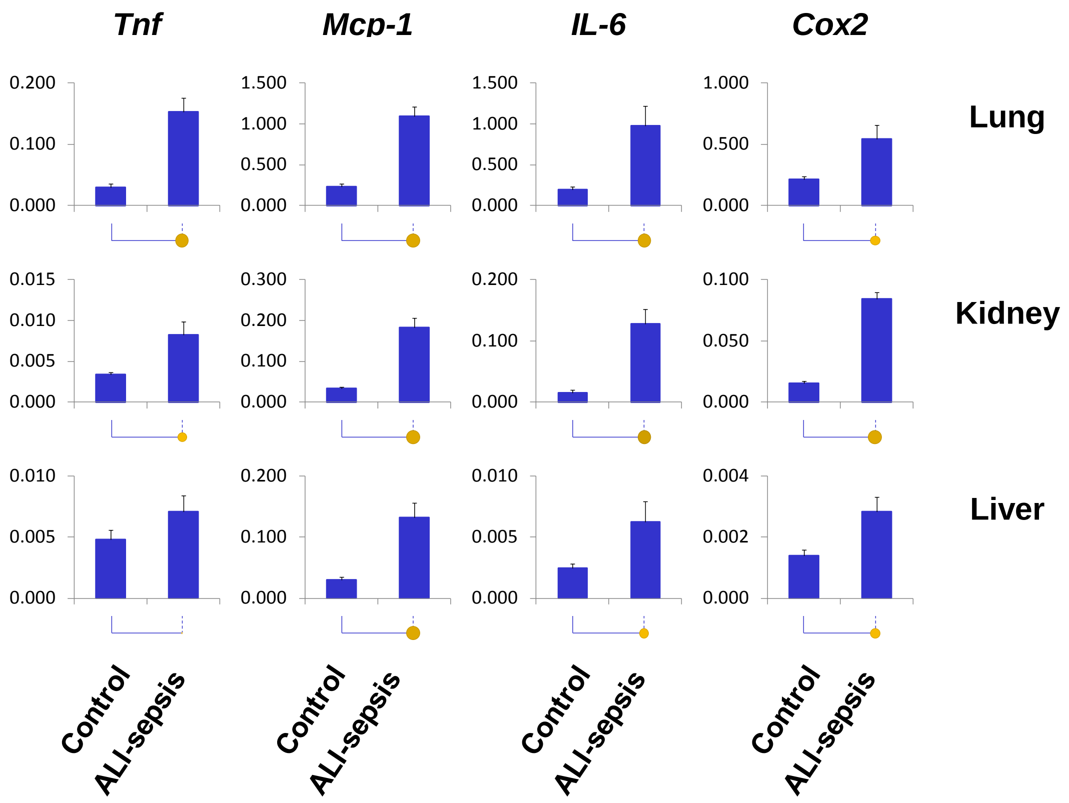

Fig.S7. Analysis of changes in lung, kidney and liver of *Tnf*, *Mpc-1*, *IL-6* and *Cox2* mRNA expression in experimental ALI-sepsis. cDNA was used in real time PCR with gene specific primers. mRNA level of a given gene in each sample was normalized to β–actin transcript. Data are represented as mean ± SEM, n= 12 mice in each group. Statistical differences between two means (p value) are shown by the size of the solid circles: $p<0.05$ for small circle, $p<0.01$ for large circle, and no circle indicating the differences are not statistically significant.
